# Both Enhanced Biocompatibility and Antibacterial Activity in Ag-Decorated TiO_2_ Nanotubes

**DOI:** 10.1371/journal.pone.0075364

**Published:** 2013-10-04

**Authors:** Ming-Ying Lan, Chia-Pei Liu, Her-Hsiung Huang, Sheng-Wei Lee

**Affiliations:** 1 Department of Otolaryngology, Taipei Veterans General Hospital, Taipei, Taiwan; 2 Institute of Clinical Medicine, National Yang-Ming University, Taipei, Taiwan; 3 Institute of Materials Science and Engineering, National Central University, Jhongli, Taiwan; 4 Department of Dentistry, National Yang-Ming University, Taipei, Taiwan; University of Akron, United States of America

## Abstract

In this study, Ag is electron-beam evaporated to modify the topography of anodic TiO_2_ nanotubes of different diameters to obtain an implant with enhanced antibacterial activity and biocompatibility. We found that highly hydrophilic as-grown TiO_2_ nanotubes became poorly hydrophilic with Ag incorporation; however they could effectively recover their wettability to some extent under ultraviolet light irradiation. The results obtained from antibacterial tests suggested that the Ag-decorated TiO_2_ nanotubes could greatly inhibit the growth of *Staphylococcus aureus*. In vitro biocompatibility evaluation indicated that fibroblast cells exhibited an obvious diameter-dependent behavior on both as-grown and Ag-decorated TiO_2_ nanotubes. Most importantly, of all samples, the smallest diameter (25-nm-diameter) Ag-decorated nanotubes exhibited the most obvious biological activity in promoting adhesion and proliferation of human fibroblasts, and this activity could be attributed to the highly irregular topography on a nanometric scale of the Ag-decorated nanotube surface. These experimental results demonstrate that by properly controlling the structural parameters of Ag-decorated TiO_2_ nanotubes, an implant surface can be produced that enhances biocompatibility and simultaneously boosts antibacterial activity.

## Introduction

Titanium (Ti) based alloys have been widely used to fabricate implantable devices such as artificial blood vessels, and orthopedic and dental implants because of their favorable mechanical properties, corrosion resistance, and biocompatibility [1]–[3]. When exposed to air, Ti forms a titanium oxide (TiO_2_) layer on its surface (approximately 10 nm thick) acting like a ceramic with excellent biocompatibility. Once the Ti implant is inserted into the human body, the host tissues directly come into contact with the TiO_2_ layer on the implant surface. Therefore, the surface characteristics of the TiO_2_ layer dominate the biocompatibility of Ti-based implants. Recently, the interaction of nanometric scale surface topography with cells has been considered to be an increasingly important factor for tissue acceptance and cell functions [4]–[6]. Various nano-topographical modifications have been proposed to improve the cell responses to Ti-based implants. For example, TiO_2_ nanowire scaffolds fabricated by the hydrothermal reaction of alkali with Ti metal, mimicking the natural extracellular matrix in structure, have been found to enhance the adhesion and proliferation of mesenchymal stem cells (MSCs) on Ti implants [7]. In addition, highly ordered TiO_2_ nanotubes fabricated on Ti implants by electrochemical anodization have attracted considerable attention. A major advantage of anodic oxidation is the ability to precisely control the diameter and shape of the nanotubular arrays to the desired scale, meeting the demands of a specific application by precisely controlling the anodization parameters. In a number of studies on the cell responses to TiO_2_ nanotubes, nanosize effects have been shown for a variety of cells [8]–[11]. Park et al. reported that vitality, proliferation, migration, and differentiation of MSCs and hematopoietic stem cells, as well as the behavior of osteoblasts and osteoclasts were strongly affected by the nanometric scale TiO_2_ surface topography with a specific response to nanotube diameters between 15 and 100 nm [12]. Our recent study also reported the diameter-sensitive cytocompatibility of TiO_2_ nanotubes treated with supercritical CO_2_ fluid [13]. In other words, cell vitality has an extremely close relationship with the geometric factors of TiO_2_ nanotube openings.

Host tissue characteristics are also crucial for the long-term success of inserted implants. When the implant itself damages or invades epithelial or mucosa barriers, it may serve as a reservoir for microorganisms thereby predisposing to infection. Once infection occurs, bacteria tend to aggregate in a hydrated polymeric matrix to form a bio-film on the implant which is difficult for the host defense and antimicrobial therapy to destroy [14], [15]. Such implant-related infections may lead to removal of the implant, revision surgery and even amputation, all of which are associated with extremely high medical costs. It is generally accepted that the most effective method to prevent bio-film buildup on implants is to prohibit initial bacterial adhesion by making the implants antibacterial [16], [17]. A variety of chemical forms of silver (Ag) have been widely used as antibacterial agents because of their strong broad-spectrum antibacterial activity, non-cytotoxicity at suitable doses, and satisfactory stability [18]–[20]. It is believed that Ag in aqueous solution can release Ag ions which interact with the main components of bacterial cells such as DNA and proteins to cause the death of the bacterium [21], [22]. Therefore, incorporating metallic Ag into Ti-based implants provides a feasible scheme for antibacterial bio-implants.

Recently, some research groups have attempted to develop Ag-loaded TiO_2_ nanotubes as bio-implants from the viewpoint of combining enhanced biocompatibility and antibacterial activity. Das et al. reported that TiO_2_ nanotubes electrodeposited with Ag had an antibacterial activity over 99% against the growth of colonies of *Pseudomonas aeruginosa* [23]. Recently, Zhao also reported that Ag incorporated into TiO_2_ nanotubes by AgNO_3_ immersion and ultraviolet (UV) irradiation possessed the capability to prevent bacterial adhesion without obvious decline for 30 days [24]. In both of these studies, all of the TiO_2_ nanotubes had a diameter of approximately 100 nm and retained the original nanotubular structure with Ag nanoparticles incorporated into the nanotubes. Nevertheless, these Ag-loaded TiO_2_ nanotubes still showed some cytotoxicity compared to Ag-free samples. Since many studies have shown that a low concentration of released Ag ions does not cause cytotoxicity [25], [26], we hypothesized that by properly controlling the structural parameters of Ag-loaded TiO_2_ nanotubes, an implant surface could be produced that enhances biocompatibility and simultaneously boosts antibacterial activity. In this study, Ag is electron-beam evaporated to modify the topography of anodic TiO_2_ nanotubes with diameters ranging from 25 nm to 100 nm. We found that 25-nm-diameter TiO_2_ nanotubes coated with 10-nm-thickness Ag not only exhibited enhanced antibacterial activity against *Staphylococcus aureus* (*S. aureus*), but also better cell responses to human fibroblasts. In addition, this activity could be attributed to the highly irregular topography on a nanometric scale of the surface of the Ag-coated nanotubes.

## Materials and Methods

### Preparation of Ag-decorated TiO_2_ Nanotubes

Self-organized TiO_2_ nanotubes were fabricated by electrochemical anodization of Ti foil (thickness of 0.127 mm, 99.7% purity, ECHO Chemical Co. Ltd., Miaoli, Taiwan). A two-electrode electrochemical cell with Ti as the anode and platinum (Pt) as the counter electrode was used. All anodization experiments were performed in ethylene glycol electrolytes containing 0.5 wt% NH_4_F at 20°C for 90 min, and all electrolytes were prepared from reagent-grade chemicals and deionized water. Anodization voltages were adjusted to result in TiO_2_ nanotubes with diameters of 25, 50, and 100 nm. Subsequently, a 10-nm-thick Ag layer was electron-beam evaporated on these nanotubes. During the deposition of Ag, the vacuum level and deposition rate were maintained at 2×10^7^ Torr and 0.1 nm/s, respectively. For the in-vitro experiments, low-intensity UV light irradiation (<2 mW/cm^2^) was used on all nanotube samples using fluorescent black-light bulbs for 8 h.

### Material Characterization

Field emission scanning electron microscopy (FE-SEM; FEI Quanta 200 F, FEI, Hillsboro, OR, USA) was employed to characterize the surface morphology of the Ag-decorated nanotubes. X-ray diffraction (XRD; D2 Phaser, Bruker, Billerica, MA, USA) and transmission electron microscopy (TEM; JEM-2100, JEOL, Japan) in conjunction with an energy dispersion spectrometer (EDS) were utilized to determine the TiO_2_ crystalline structure and Ag distribution in the nanotubes. The surface wettability of the materials was evaluated by measuring the contact angle between the TiO_2_ nanotubes and water droplets in the dark. Contact angle measurements were performed at room temperature by the extension method using a horizontal microscope with a protractor eyepiece.

### Ag Ion Release

The amount of Ag released from the Ag-decorated TiO_2_ nanotubes was monitored in phosphate buffered saline (PBS) at 37°C. The Ag-decorated nanotubes were immersed in 10 ml of PBS for 1 day in the dark, taken out, and then immersed again in 10 ml of fresh PBS. This process was repeated for a total of 14 days to generate solutions at different time points in order to obtain the Ag release time profile. The PBS solution containing the released Ag ions was analyzed by inductively-coupled plasma mass spectrometry (ICP-MS; ELAN 6100, Perkin-Elmer, Waltham, MA, USA). The accumulative amounts of Ag ion release were presented in this study.

### Human Fibroblast Cell Culture

MRC-5 human fibroblasts were purchased from the Bioresource Collection and Research Center, Taiwan. The cells were plated in a 10-cm tissue culture plate and cultured with Eagle’s minimum essential medium (Gibco, Life Technologies Corporation, Grand Island, NY, USA) containing 10% fetal bovine serum, 2 mM L-glutamine, 1.5 g/L sodium bicarbonate, 0.1 mM non-essential amino acids, and 1.0 mM sodium pyruvate. Cultures were maintained at 37°C in a humidified atmosphere of 5% CO_2_. The cells were then seeded onto the autoclaved nanotube samples placed on the bottom of 12-well culture plate (Falcon, BD Biosciences, San Jose, CA, USA) at a density of 1×10^4^ cells/cm^2^ for 3 days for cell adhesion and proliferation assay.

### Cell Adhesion Assay

For cell adhesion experiments, 3 days after cell plating, non-adherent cells were washed with PBS. The adhered cells were fixed in 4% paraformaldehyde (USB Corp., Cleveland, OH, USA) at room temperature for 1 h and washed with PBS. After fixation, the cells were permeabilized with 0.1% Triton X-100 (Sigma-Aldrich Corporation, St. Louis, MO, USA) in PBS for 15 min at 4°C. The cells were then washed with PBS and incubated with rhodamine phalloidin (Life Technologies Corporation, Grand Island, NY, USA) for 15 min for actin filament staining, and with diamidino-2-phenylindole (DAPI; Thermo Fisher Scientific Inc., Waltham, MA, USA) for 5 min for nuclei staining. Images of the stained fibroblasts were taken using a fluorescence microscope to examine the cell adhesion and cytoskeletal arrangement. For SEM observation, the cells were fixed with 2.5% glutaraldehyde solution (Merck & Co., Inc., Whitehouse Station, NJ, USA) for 1 h at room temperature. The samples were rinsed in PBS solution twice, dehydrated in a series of ethanol solutions (40, 50, 60, 70, 80, 90, and 100% v/v) and critical point dried with a critical point dryer (CPD 030, Leica Microsystems, Wetzlar, Germany). A thin Pt film was coated onto the samples before SEM observation.

### Cell Proliferation Assay

Cell viability was determined using a WST-1 cell proliferation reagent kit (Roche, Woerden, Netherlands) according to the manufacturer’s instructions. On the 3rd day, cells on the nanotubes were washed with PBS twice, and then incubated with a medium containing 10% WST-1 cell proliferation reagent at 37°C in a humidified atmosphere of 5% CO_2_ for 2 h. The solution was then retrieved from each well and plated in a 96-well plate, and optical densities were measured using a spectrophotometer (Tecan Group Ltd., Männedorf, Switzerland) at 450 nm.

### Protein Adsorption

To evaluate the adsorption of proteins on the Ag-decorated TiO_2_ nanotubes, bovine serum albumin (BSA; Sigma-Aldrich Corporation, St. Louis, MO, USA) was used as a model protein. X-ray photoelectron spectroscopy (XPS) was employed to analyze the nitrogen spectra (in terms of N 1 s) on the nanotube surfaces after 5 min of immersion in BSA solution (5 mg BSA in 1 ml PBS).

### Antibacterial Test

The antibacterial activity against *S. aureus* (ATCC 25923, Bioresource Collection and Research Center, Hsinchu, Taiwan) was then evaluated. *S. aureus* was cultured in tryptic soy broth and agar plates. The nanotube samples were sterilized in an autoclave at 121°C for 40 min, and then a suspension containing the bacteria at a concentration of 4×10^9^ cfu ml^−1^ was introduced onto the surface of the samples to a density of 150 µl cm^−2^. The samples with the bacterial suspension were incubated at 37°C for 4 h. Subsequently, the samples were rinsed with sterile water and bacteria were detached by sonication from the surface of the nanotubes. The bacteria solution was collected and diluted 100X before being plated on an agar plate. After incubation at 37°C for 24 h, the active bacteria were counted and the agar plates were photographed. The examination of the morphology of the bacteria adhering to the nanotube surface was performed by SEM. The samples were prepared by plating a 2×10^10^ cfu ml^−1^ suspension on the sample surface with a density of 150 µl cm^−2^ followed by incubation at 37°C for 4 h. At the end of incubation, the loosened and detached bacterial cells were removed by dipping the samples into sterile PBS. Subsequently, the bacteria adhering to the surface were first fixed by 2.5% glutaraldehyde solution (Merck & Co., Inc., Whitehouse Station, NJ, USA) for 1 h at room temperature. Then the samples were dehydrated in a graded series of ethanol solutions (40, 50, 60, 70, 80, 90, and 100% v/v) and critical point dried with a critical point dryer (CPD 030, Leica Microsystems, Wetzlar, Germany). A thin Pt film was coated onto the samples before SEM observation.

### Statistical Analysis

All experiments were carried out in triplicate and at least three independent experiments were performed. The test values were expressed as mean ± standard error (SE). In Ag ion release experiment, Student *t* test was used to compare Ag ion concentration between 25- and 100-nm-diameter groups. In cell proliferation assay, statistic comparisons of multi-group data were analyzed by ANOVA, followed by Scheffe’s post-test using SPSS 12.0 software (SPSS Inc., Chicago, IL). A *p* value of less than 0.05 was considered to be statistically significant.

## Results and Discussion

**Figure 1:**
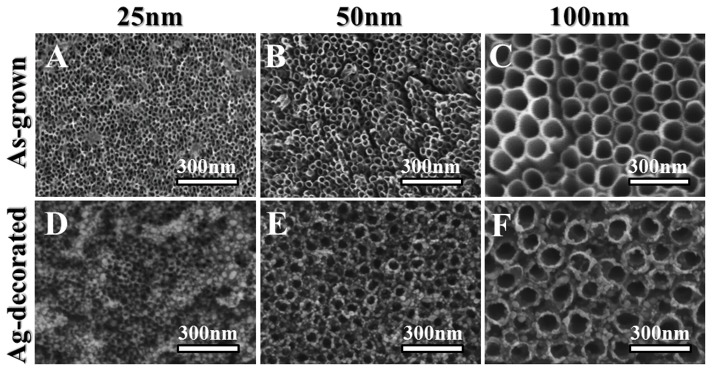
SEM images of self-organized TiO_2_ nanotubes with different diameters. The as-grown (**A**–**C**) and Ag-decorated (**D**–**F**) nanotubes had diameters of 25, 50, and 100 nm, respectively.

Figure 1A–C shows the SEM images of the as-anodized TiO_2_ nanotubes with diameters of 25 nm, 50 nm, and 100 nm, produced by electrochemical anodization at the applied voltages of 10 V, 20 V, and 40 V, respectively. These as-grown TiO_2_ nanotubes had well-defined nanotubular structure, and their nanotube diameters were nearly proportional to the applied voltages. In addition, the XRD results from our previous study confirmed these as-grown TiO_2_ nanotubes to be amorphous phase, mainly TiO_2_•xH_2_O [13]. After the Ag deposition, the surface topography of these as-grown TiO_2_ nanotubes changed with the extent depending on the diameter. For the 100-nm-diameter sample (Figure 1F), the original nanotubular structure was almost completely retained except that the tube opening slightly shrank due to the decoration of the Ag nanoparticles. The influence of Ag decoration on the nanotube topography became more significant with a deceasing nanotube diameter (Figure 1D and 1E). For the smallest diameter (25 nm) nanotubes, some openings were fully covered with Ag nanoparticles, resulting in more irregular topography on a nanometric scale.

**Figure 2:**
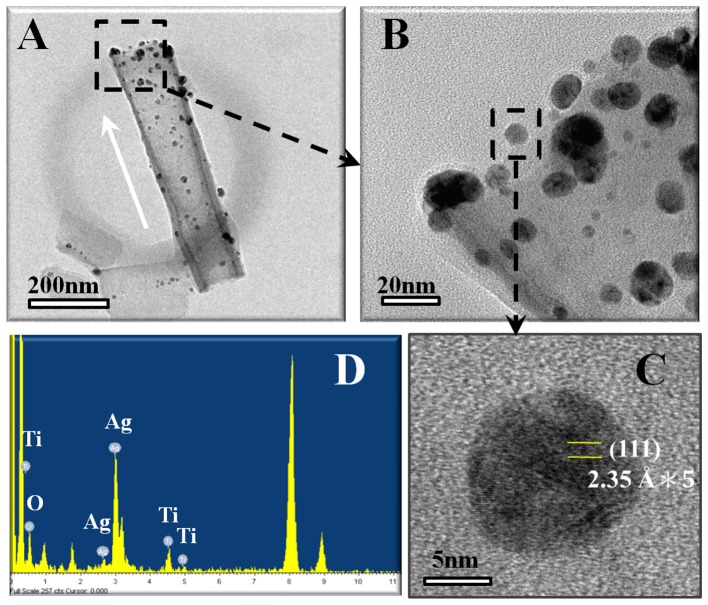
TEM analysis results of an Ag-decorated TiO_2_ nanotube. (**A**) TEM image taken from an Ag-decorated TiO_2_ nanotube with the diameter of 100 nm. (**B**, **C**) High-magnification views of the selected regions, and (**D**) the corresponding EDS spectrum. The white arrow in (**A**) indicates the growth direction of the TiO_2_ nanotube.

**Figure 3:**
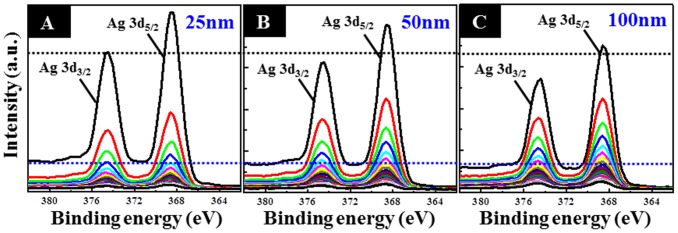
High-resolution XPS spectra in terms of Ag 3d at different depths. For the Ag-decorated TiO_2_ nanotubes with the diameters of (**A**) 25, (**B**) 50, and (**C**) 100 nm.

Figure 2 shows the TEM analysis results for a 100-nm-diameter Ag-decorated TiO_2_ nanotube. Based on the atomic plane spacing in high-magnification TEM images (Figure 2B and 2C) and EDS spectrum (Figure 2D), metallic Ag nanoparticles were confirmed to be loaded into the TiO_2_ nanotube. The Ag nanoparticles were mainly distributed near the nanotube surface, while some were incorporated into the inner surface of the nanotubes (Figure 2A). This scenario is reasonable since Ag nanoparticles have a particle size ranging from 5 to 20 nm, which is much smaller than the nanotube opening. The XPS spectra at different depths using a constant Ar^+^ sputtering rate (Figure 3) further compared the Ag distribution in the TiO_2_ nanotubes of different diameters. The binding energies of the Ag 3d peak at 368.25 and 374.25 eV could be assigned to 3d_5/2_ and 3d_3/2_ of metallic Ag^0^, respectively [27], indicating that the Ag nanoparticles existed in the Ag^0^ state in the TiO_2_ nanotubes. We found that, compared to the nanotubes with larger diameters, the 25-nm-diameter Ag-decorated nanotubes showed a relatively strong Ag signal near the surface, indicating that the Ag nanoparticles had mainly aggregated near the nanotube surface and thus caused the more irregular topography seen in Figure 1D.

**Figure 4:**
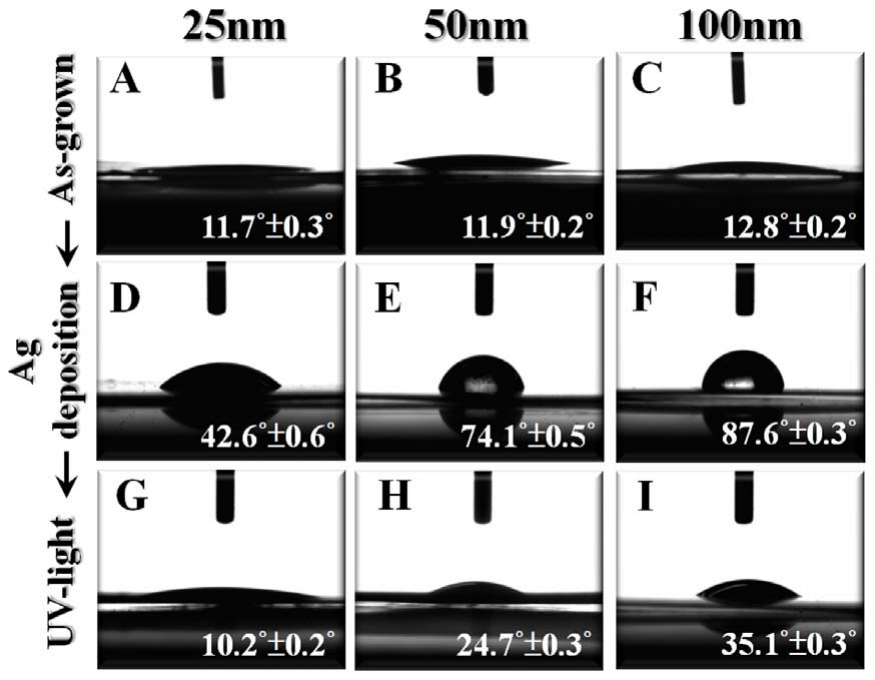
Optical images showing water droplets. On the as-grown (**A**–**C**) and Ag-decorated TiO_2_ nanotubes before (**D**–**F**) and after UV light irradiation (**G**–**I**). The contact angles are denoted in the images. Data were means ±SE of three independent experiments.

It has been reported that cell attachment, spread, and cytoskeletal organization are apparently greater on hydrophilic relative to hydrophobic surfaces [28]. Das et al. also indicated that a low contact angle means high surface energy, which is a crucial factor contributing to better cell attachment [29]. It was thus essential to study the surface wettability of the Ag-decorated TiO_2_ nanotubes. In our previous study, all as-grown TiO_2_ nanotubes with different diameters were highly hydrophilic as their contact angles were quite small (Figure 4A–C). Nevertheless, after the Ag-decoration process, these nanotube samples became poorly hydrophilic and their contact angles increased with an increasing diameter (Figure 4D–F). This phenomenon can be explained by Wenzel’s model [30], in which an increase of surface roughness in hydrophilic material will result in a smaller contact angle, and water will fill the grooves below the droplet. Hence larger diameter nanotubes, having smaller geometric roughness, are thought to exhibit poorer hydrophilicity. However, once irradiated with UV-light for 1 h, the Ag-decorated TiO_2_ nanotubes recovered their hydrophilicity to an extent depending on the diameter (Figure 4G–I). TiO_2_ is a photosensitive material, and when irradiated with UV light the photo-generated holes react with lattice oxygen to form surface oxygen vacancies to which water molecules kinetically coordinate, thereby greatly improving the surface hydrophilicity. In particular, the Ag-decorated TiO_2_ materials in this study provided better separation between electrons and holes [31], further promoting the generation of oxygen vacancies and surface wettability.

**Figure 5:**
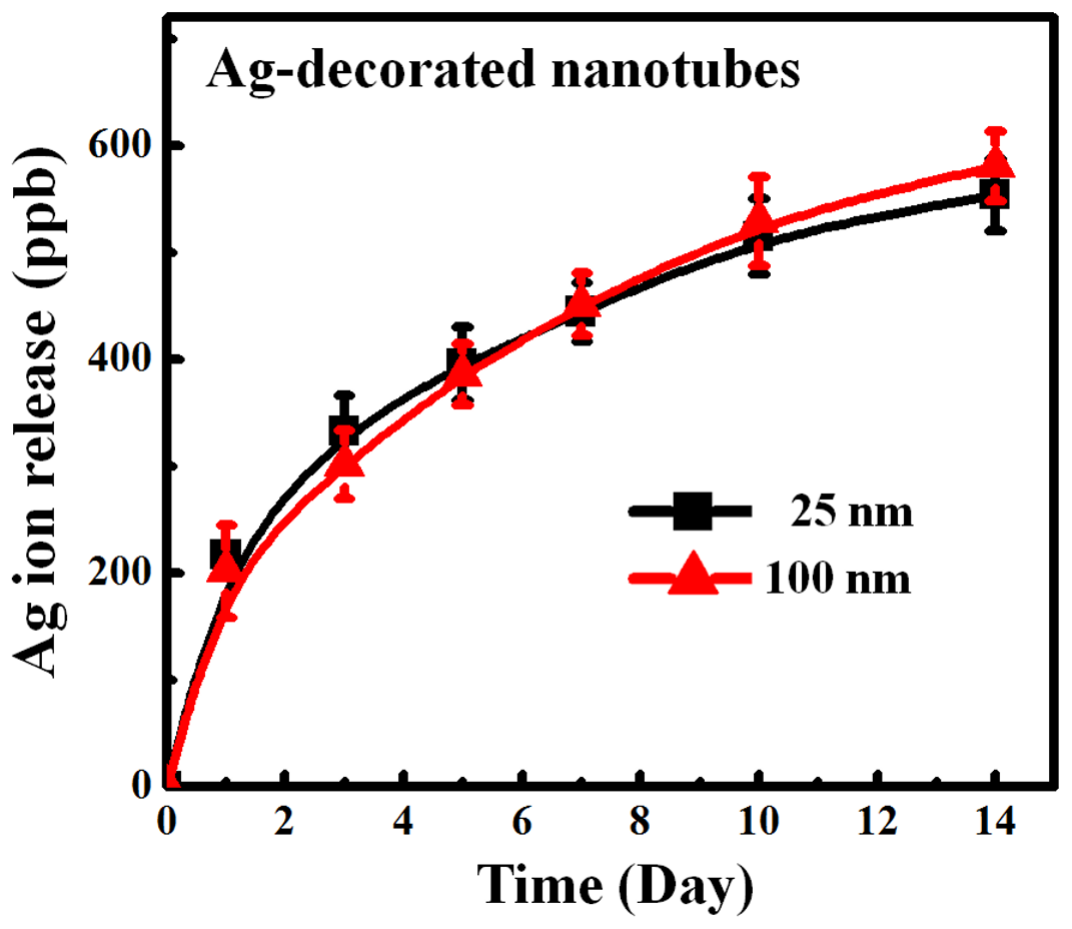
Cumulative Ag release profiles from the 25- and 100-nm-diameter Ag-decorated nanotubes into PBS. There were no statistical differences among the Ag-decorated samples with regards to the nanotube diameter (*p>0.05*). Data were means ±SE of three independent experiments.

Figure 5 shows the cumulative Ag ion release profiles from the Ag-decorated nanotubes of different diameters into PBS solution. Both the 25- and 100-nm-diameter Ag-decorated nanotubes initially showed a higher Ag ion release rate, with a gradual decrease in release with immersion time. However, there were no statistical differences among the Ag-decorated samples with regards to the nanotube diameter. The initial phase after implantation is the most dangerous and prone to infection, and a higher Ag ion release rate in the early stage is required to avoid post-operative infections and guarantee normal wound healing. Hence the Ag ion release profile and rate shown in this study met the clinical requirements.

**Figure 6:**
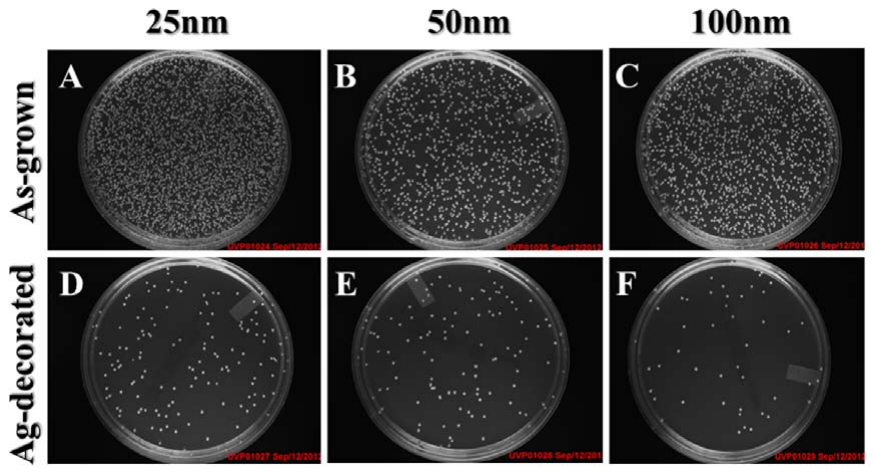
Photographs of the antibacterial activity test. Plates with *S. aureus* were grown in the presence of as-grown (**A**–**C**) and Ag-decorated (**D**–**F**) TiO_2_ nanotubes of different diameters.

**Figure 7:**
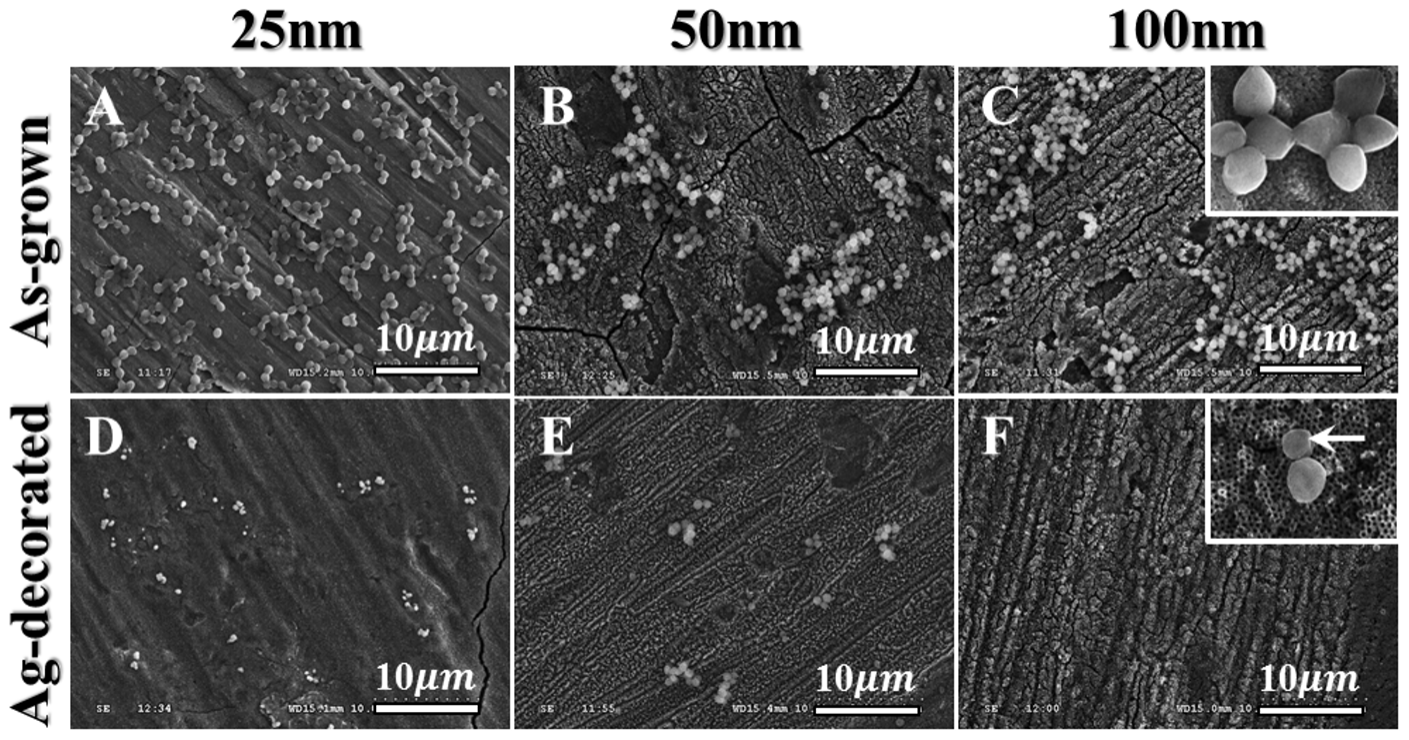
SEM images of *S. aureus* grown on as-grown or Ag-decorated TiO_2_ nanotubes of different diameters. Numerous bacteria colonies on the as-grown nanotubes (**A–C**), while only a few bacteria can be seen on the Ag-decorated series (**D–F**).

The ability of Ag-decorated TiO_2_ nanotubes to prevent viable bacteria colonization was verified by antibacterial tests as shown in Figure 6. The amount of viable bacteria was apparently smaller on the Ag-decorated samples compared to the as-grown samples (Figure 6D–F). This result indicates that the decoration of Ag nanoparticles on TiO_2_ nanotubes effectively inhibited the growth of bacterial colonies and showed enhanced antibacterial activity. However, there were no statistical differences among the Ag-decorated samples with regards to the diameter. SEM examinations were performed to further investigate the influence of Ag decoration of TiO_2_ nanotubes on the bacterial morphology. As shown in Figure 7, numerous bacteria tended to aggregate in groups on the as-grown nanotubes, while only a few bacteria were seen on the Ag-decorated nanotubes. In addition, the *S. aureus* displayed smooth and intact bacterial membranes on the Ag-free samples (inset in Figure 7C), while obvious membrane shrinkage and deformation were observed on the Ag-decorated samples (arrow in inset in Figure 7F). These observations indicate that Ag-decorated TiO_2_ nanotubes do indeed exhibit enhanced antibacterial activity against *S. aureus*.

The bactericidal mechanism of Ag is not fully understood, however it is generally recognized that Ag ions simultaneously attack multiple sites within the microorganism to inactivate several critical physiological functions such as cell wall synthesis, membrane transport, nucleic acid synthesis and translation, protein folding and function, and electron transport [32], [33]. It is thought that Ag ions tend to have a higher affinity to react with negatively charged side groups of the proteins on the bacterial membrane such as phosphorus and sulfur compounds [34], [35], which results in alteration of the molecular structure and destruction of the bacterial membrane [36]. Due to the destruction of the cell membrane, Ag ions are able to invade the bacteria, reacting with thiol groups of vital enzymes [37] and combining with DNA to disable the replication ability [38], and eventually causing the death of the bacteria. In addition, Ag ions can produce reactive oxygen species (ROS) which causes significant damage to cell structures [39]. The multifaceted bactericidal mechanisms of Ag may furnish Ag-decorated TiO_2_ nanotubes with a broad spectrum of antibacterial activity.

**Figure 8:**
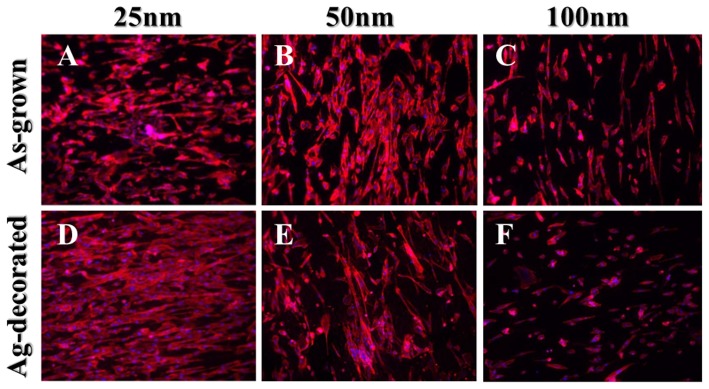
Fluorescence images of the human fibroblast cells attached on the as-grown (A–C) and Ag-decorated (D–F) TiO_2_ nanotubes of different diameters. The red fluorescence indicates cytoskeletal actin, and the blue fluorescence indicates cell nuclei. For both as-grown and Ag-decorated TiO_2_ nanotubes, longer, better-defined actin cytoskeleton, and higher density of fibroblasts were noted on the smaller diameter nanotubes.

**Figure 9:**
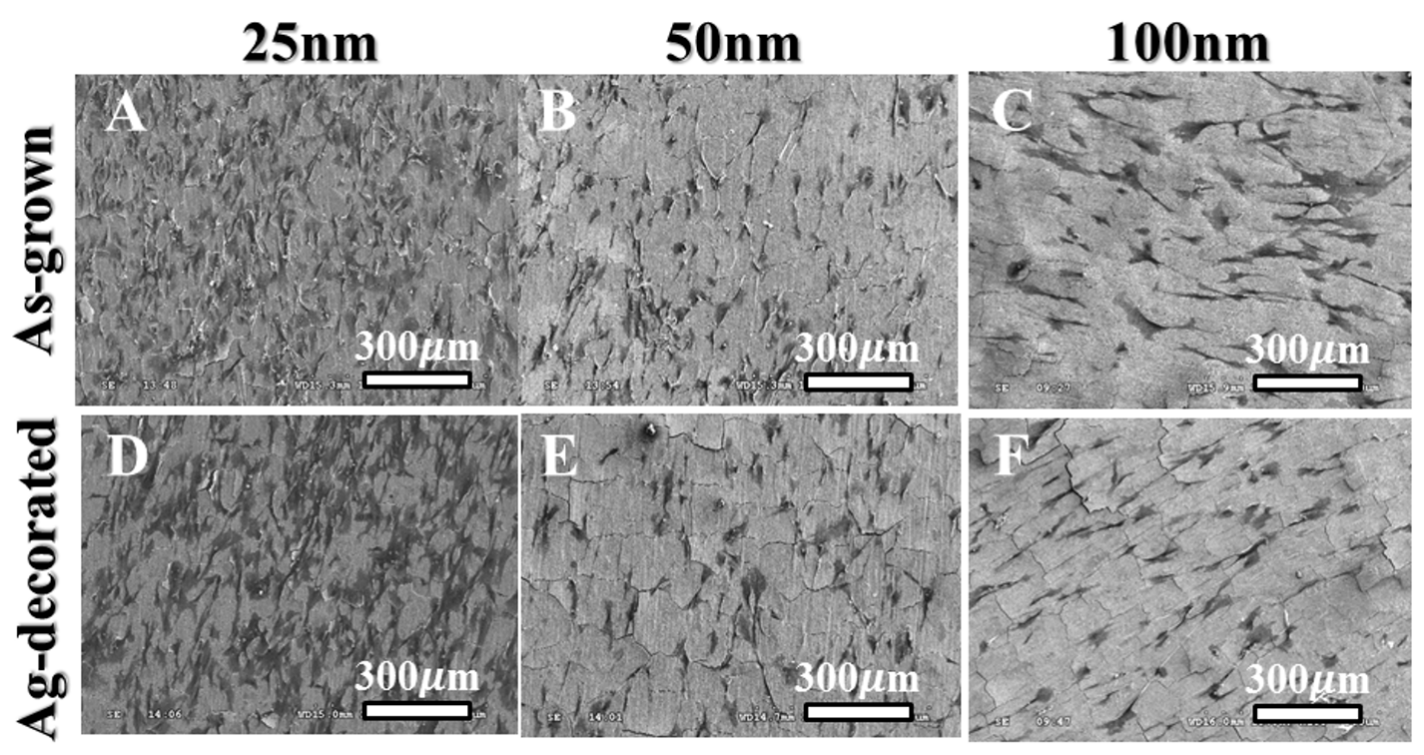
SEM images showing the cell adhesion and proliferation of human fibroblast cells on the as-grown (A–C) and Ag-decorated (D–F) TiO_2_ nanotubes with the diameters of 25, 50, and 100 nm, respectively. The fibroblasts cultured on the 25-nm-diameter nanotubes revealed good cell adhesion with development of an elongated cytoskeleton, while those on the 50-nm-diameter or larger nanotubes show rounded cell morphology with a lack of cell spread in both the as-grown and Ag-decorated samples.

The human fibroblast cell behavior in response to the Ag-decorated TiO_2_ nanotubes was studied, since the issue of cytotoxicity is crucial for practical implantations. To evaluate the fibroblast cell attachment on the TiO_2_ nanotubes, cytoskeleton actin was stained with rhodamine phalloidin which expressed red fluorescence, and the nuclei were stained with DAPI which expressed blue fluorescence. The actin immunostaining showed very different cell-material contact morphology for the nanotube samples with different diameters (Figure 8). For both as-grown and Ag-decorated nanotubes, and especially the Ag-decorated series, there were much longer and well-defined actin fibers on the fibroblasts cultured on 25-nm-diameter nanotubes relative to the larger ones. It is known that cells have to adhere to a material surface first and then spread for further cell division. Better cell adhesion leads to more activation of intracellular signaling cascades through integrin coupled to the actin cytoskeleton [40], [41], and hence the smaller diameter nanotubes, even those decorated with Ag nanoparticles, had more focal points for the fibroblast cells to get attached and thus aid in cell adhesion. FE-SEM was employed for detailed observations of cell adhesion (Figure 9). The fibroblasts on the 25-nm-diameter nanotubes revealed good cell adhesion with an elongated flattened morphology, while those on the 50-nm-diameter or larger nanotubes show more rounded morphology and lack of cell spreading. It has also been reported that cells recognize surface features when a suitable site for adhesion has been detected. The cells then stabilize the contact by forming focal adhesions and mature actin fibers, followed by the recruitment of tubulin microtubules [40]. The actin cytoskeleton has been linked to integrins which are located within the adhesions. Our findings suggest that the cytoskeleton on the smaller diameter nanotubes, even those decorated with Ag nanoparticles, was formed more successfully than that on the larger diameter nanotubes. It should be noted that the aforementioned difference in surface wettability may also have contributed to and even enhanced the difference in cell adhesion between the Ag-decorated samples.

**Figure 10:**
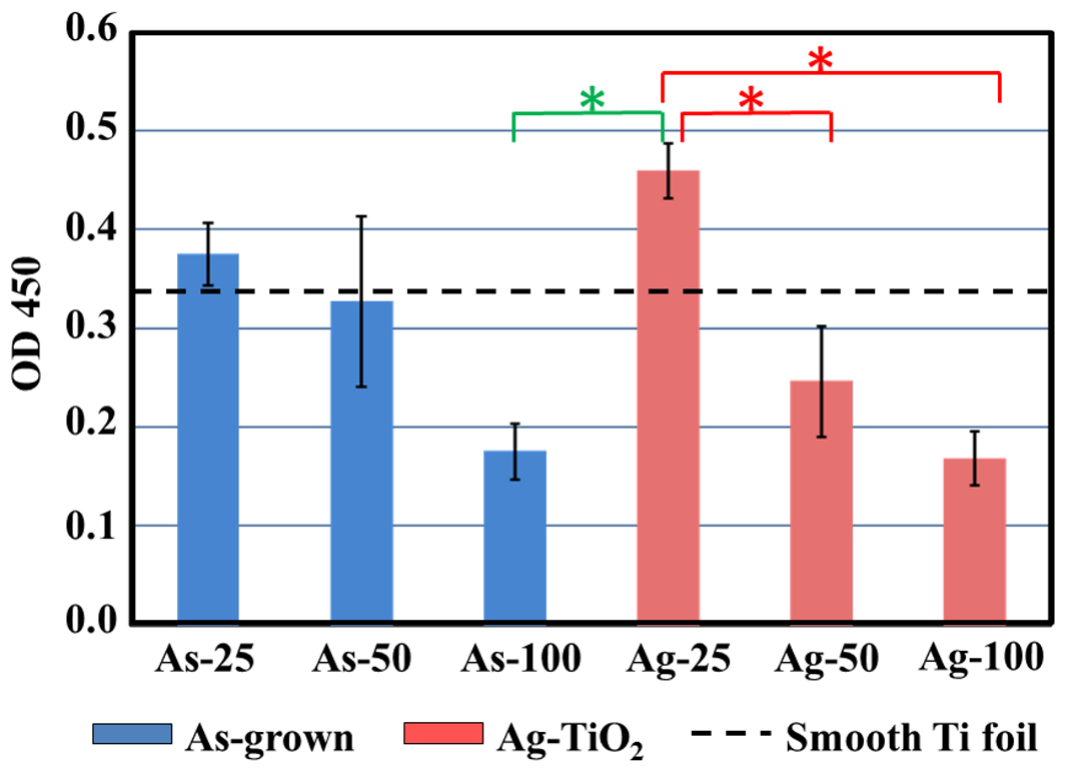
Optical densities (OD) measured after the culture of human fibroblast cells on the as-grown and Ag-decorated TiO_2_ nanotubes of different diameters. Cell proliferation was lowest for the largest diameter of 100-grown and Ag-decorated TiO_2_ nanotubes. Meanwhile, the 25-nm-diameter Ag-decorated sample showed the highest OD value of all nanotube samples. Data were means ±SE of three independent experiments (*p<0.01).

To further quantitatively evaluate the cell proliferation on the TiO_2_ nanotubes, the WST-1 assay was used after cell seeding on the samples for 3 days. Figure 10 shows the comparison of optical densities measured from the WST-1 assay. We found that both as-grown and Ag-decorated nanotubes exhibited a monotonically increasing trend in cell proliferation with decreasing nanotube diameter, indicating that the fibroblast cells showed an obvious diameter-dependent behavior on both as-grown and Ag-decorated nanotubes. We also found that cell proliferation on the smallest diameter nanotubes was enhanced in comparison to the plain Ti foil. Further, the 25-nm-diameter Ag-decorated samples showed a higher optical density compared to their as-grown counterparts. This result not only indicates that the Ag nanoparticles do not exhibit cytotoxicity through an appropriate release mechanism, but also that by properly controlling the structural parameters of Ag-loaded TiO_2_ nanotubes, both enhanced biocompatibility and antibacterial activity can be achieved. It has been reported that the predicted size of surface occupancy by the head of an integrin heterodimer composed of a β-propeller of the α-chain and the A domain of the β-chain is about 10 nm as estimated from electron micrographic images of individual integrin molecules [42]. It has been suggested that a 15–25 nm spacing facilitates clustering of integrins into nearly the closest packing possible, resulting in optimal integrin activation. We speculate that the decoration of Ag nanoparticles modified the surface topography of the 25-nm-diameter nanotubes, causing more irregular topography on a nanometric scale. Such a highly irregular topography may provide more suitable nanometric sites for integrin clustering, thus resulting in enhanced cell proliferation. On the other hand, nanotube diameters larger than 50 nm, which almost retained the original tubular size with the Ag incorporation, severely hindered cell spreading, adhesion, and completely prevented integrin clustering and the formation of focal adhesion complexes, eventually resulting in dramatically reduced cell proliferation. Recently, Zhao developed hybrid poly(*N*-hydroxyethylacrylamide) (polyHEAA)/salicylate (SA) hydrogels that combine the advantages of both antifouling and antimicrobial materials [43]. Such dual functional hydrogels, exhibiting high surface resistance to both cell adhesion and bacteria attachment, hold great potential for biomedical applications such as wound dressing and other self-healing materials, while the Ag-decorated TiO_2_ nanotubes fabricated in the present study that enhance biocompatibility and simultaneously boost antibacterial activity could be potential promising materials for medical implants.

**Figure 11:**
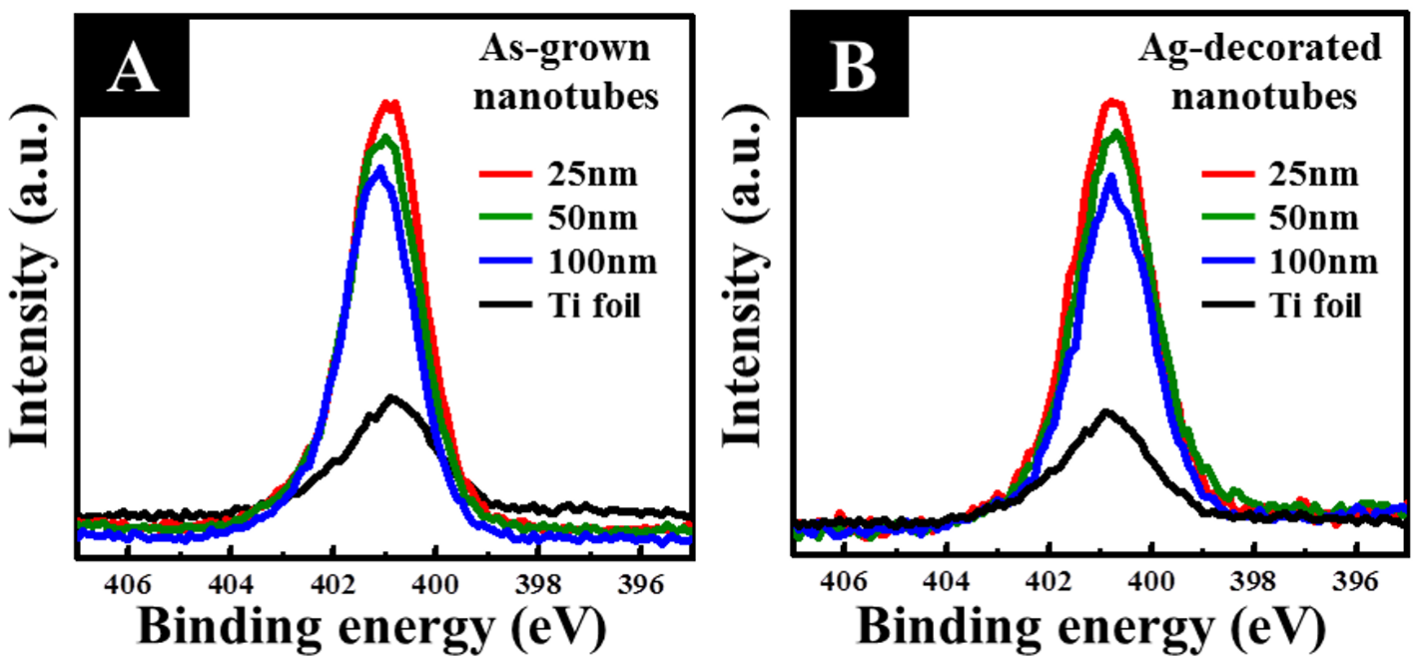
XPS surface analysis results, in terms of spectra for N 1s of the as-grown and Ag-decorated TiO_2_ nanotubes. All nanotube samples of different diameters, either as-grown or Ag decorates series, had much higher N 1 s intensity than the plain Ti foil.

Figure 11 further shows the XPS surface analysis results, in terms of spectra for N 1 s, of all samples after immersion in the cell culture medium for 5 minutes. It shows that all of the nanotube samples with different diameters, either as-grown or Ag-decorated, had much higher N 1 s intensity than the plain Ti foil. In other words, a higher protein content existed on the nanotube samples relative to the plain Ti foil. However, there were no statistical differences among the as-grown and Ag-decorated nanotube samples. This also indicates that the decoration of Ag nanoparticles had no negative effects on the protein adsorption ability of the TiO_2_ nanotubes. It has been shown that increased protein adsorption promotes osteoblastic adhesion via an enhanced interaction between cellular integrins and proteins [44]. This would promote all of the cell responses, including cell adhesion, cell spreading, and cell proliferation.

These results demonstrate that decorating TiO_2_ nanotubes with Ag is a feasible scheme for fabricating implants that exhibit antibacterial activity and enhanced biocompatibility. In particular, the stability of the Ag-decorated TiO_2_ structure in the physiological environment is excellent because of its inorganic nature. Most importantly, the fabrication process for Ag-decorated TiO_2_ nanotubes is easy, low-cost, and suitable for industrial production. We also believe that the biocompatibility can be further tailored by optimizing the structural parameters or modifying the fabrication process of Ag-decorated TiO_2_ nanotubes. For example, Ag nanoparticles can be incorporated into TiO_2_ nanotubes by immersion in AgNO_3_ solution followed by UV light radiation. Process parameters such as the AgNO_3_ concentration, immersion time, and additives can be optimized to increase the loading amount of incorporated Ag. In addition, nanometric Ag nanoparticles with adjustable sizes and shapes can be synthesized [45] and then incorporated into the TiO_2_ nanotubes more effectively to yield long-lasting antibacterial effects. These experiments in our laboratory are still ongoing.

## Conclusions

In this study, Ag is electron-beam evaporated to modify the topography of anodic TiO_2_ nanotubes of different diameters to obtain an implant with enhanced antibacterial activity and biocompatibility. We found that highly hydrophilic as-grown TiO_2_ nanotubes became poorly hydrophilic with the decoration of Ag nanoparticles; however they could effectively recover their wettability to some extent after UV light irradiation. The results obtained from antibacterial tests suggested that the Ag-decorated TiO_2_ nanotubes could greatly inhibit the growth of *S. aureus*. In vitro biocompatibility evaluation indicated that fibroblast cells exhibited an obvious diameter-dependent behavior on both as-grown and Ag-decorated TiO_2_ nanotubes. Most importantly, of all samples, the smallest diameter (25 nm) Ag-decorated nanotubes exhibited the most obvious biological activity in promoting adhesion and proliferation of human fibroblasts, and this activity could be attributed to the highly irregular topography on a nanometric scale of the Ag-decorated nanotube surface. These experimental results demonstrate that by properly controlling the structural parameters of Ag-loaded TiO_2_ nanotubes, an implant surface can be produced that enhances biocompatibility and simultaneously boosts antibacterial activity.
